# Recent Advances in Effector-Triggered Immunity in Plants: New Pieces in the Puzzle Create a Different Paradigm

**DOI:** 10.3390/ijms22094709

**Published:** 2021-04-29

**Authors:** Quang-Minh Nguyen, Arya Bagus Boedi Iswanto, Geon Hui Son, Sang Hee Kim

**Affiliations:** 1 Division of Applied Life Science (BK21 Four Program), Plant Molecular Biology and Biotechnology Research Center, Gyeongsang National University, 501 Jinju-daero, Jinju 52828, Korea; nguyenquangminh4595@gmail.com (Q.-M.N.); aryabagus62@gmail.com (A.B.B.I.); geonhees@gnu.ac.kr (G.H.S.); 2 Division of Life Science, Gyeongsang National University, 501 Jinju-daero, Jinju 52828, Korea

**Keywords:** pathogen, effector, PTI, ETI, NLR, plant immunity

## Abstract

Plants rely on multiple immune systems to protect themselves from pathogens. When pattern-triggered immunity (PTI)—the first layer of the immune response—is no longer effective as a result of pathogenic effectors, effector-triggered immunity (ETI) often provides resistance. In ETI, host plants directly or indirectly perceive pathogen effectors via resistance proteins and launch a more robust and rapid defense response. Resistance proteins are typically found in the form of nucleotide-binding and leucine-rich-repeat-containing receptors (NLRs). Upon effector recognition, an NLR undergoes structural change and associates with other NLRs. The dimerization or oligomerization of NLRs signals to downstream components, activates “helper” NLRs, and culminates in the ETI response. Originally, PTI was thought to contribute little to ETI. However, most recent studies revealed crosstalk and cooperation between ETI and PTI. Here, we summarize recent advancements in our understanding of the ETI response and its components, as well as how these components cooperate in the innate immune signaling pathways. Based on up-to-date accumulated knowledge, this review provides our current perspective of potential engineering strategies for crop protection.

## 1. Introduction

**Figure 1:**
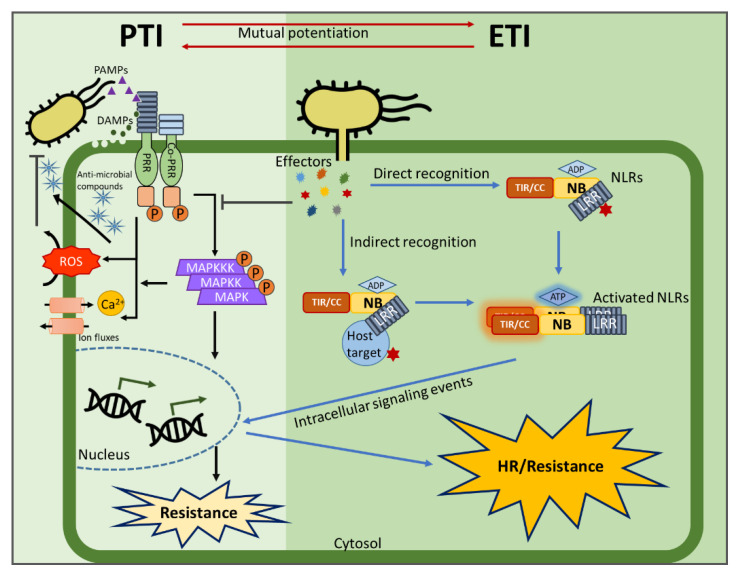
Schematic view of pattern-triggered immunity (PTI) and effector-triggered immunity (ETI) in plants. The first layer of induced immunity, called PTI (indicated by black arrows), is activated by the recognition of pathogen-associated molecular patterns (PAMPs) or damage-associated molecular patterns (DAMPs) through pattern recognition receptors (PRRs). Several PTI signaling events occur, such as activation of the mitogen-activated protein kinases (MAPK) kinase cascades, an influx of Ca^2+^ into the cytosol, and production of reactive oxygen species (ROS). Antimicrobial compounds are produced and the defense genes are activated. However, to suppress PTI, the pathogens deploy effectors. When they are recognized by nucleotide-binding (NB) and leucine-rich-repeat (LRR)-containing receptors (NLRs), the second immune layer, called ETI (indicated by blue arrows), takes place. NLRs directly or indirectly perceive pathogenic effectors, leading to a conformational change, which together with several intracellular signaling events, ultimately trigger the hypersensitive response (HR) or other defense responses. Surprisingly, the most recent studies reported that PTI and ETI are mutually linked and together potentiate the immune response (indicated by red arrows).

Plants and pathogens continually compete for supremacy as they coevolve. In nature, many plants are resistant to most pathogens, but some pathogenic microbes are capable of causing severe diseases. The primary barrier of plants against pathogenic invasion is the preformed defense layer, including the plant cell wall and pre-produced metabolites [1,2,3,4]. To successfully respond to and defend against pathogenic microbes, plants developed multilayered protective and surveillance networks [5]. The first layer of the plant immune system is pattern-triggered immunity (PTI), which is activated by pathogen-associated molecular patterns (PAMPs), the conserved molecular structures of pathogens such as fungal chitin or bacterial flagellin, or damage-associated molecular patterns, which are molecules resulting from plant–pathogen interactions such as peptides and oligosaccharides (Figure 1). These inducers can be recognized by pattern recognition receptors (PRRs), plasma membrane-localized plant immune receptors, which are mainly found in the forms of receptor-like protein kinases and receptor-like proteins [6,7,8]. Activation of these receptors provokes an array of plant defense responses to halt pathogen spread and colonization [9,10]. PTI activates multiple signaling pathways in the host cells (Figure 1). One of the rapid responses is an influx of extracellular Ca^2+^ into the cytosol [11,12,13], followed by the activation of mitogen-activated protein kinases [14,15], reactive oxygen species (ROS) signaling [12,16], and other signaling molecules, such as reactive nitrogen species, lipids, callose, salicylic acid, n-hydroxypipecolic acid, jasmonic acid, ethylene, and cytokinin [17,18,19,20,21,22,23,24,25,26,27,28,29]. However, to defeat PTI responses, many pathogens deploy a variety of effector proteins (Figure 1). When they are recognized by specialized receptors in the plant called resistance (R) proteins, the second layer of plant immune responses is activated, which is effector-triggered immunity (ETI) [30,31].

Structural and functional analysis in current studies of many R proteins reveals that there are two conserved features, nucleotide-binding (NB) and leucine-rich repeat (LRR) domains, known as NLRs. The structure of other NLR domains depends on whether a Toll-interleukin 1-like receptor (TIR) or a coiled-coil (CC) is attached at the N terminus (Figure 1) [32,33]. Host plants employ a diverse family of NLRs to detect effectors rapidly during pathogen invasion. NLRs selectively recognize the effectors, either directly or indirectly, and such recognition often leads to a hypersensitive response, a form of rapid localized programmed cell death (Figure 1) [28,34,35]. The immune responses elicited by PRRs and NLRs are similar, although the duration and amplitude of ETI responses are often vastly larger than those of PTI responses (Figure 1) [30]. However, it was reported that there is a hefty overlap in the transcriptional regulation during PTI and ETI [36,37]. Surprisingly, the most recent studies reported that there is even a substantial linkage between NLR-mediated ETI and PRR-mediated PTI (Figure 1) [38,39].

Genetic and molecular approaches successfully discovered and identified several NLRs required for the ETI signaling networks. Indeed, both approaches contributed significantly to the study of plant immunity. Here, we summarize the current understanding and provide a perspective of crop engineering strategies based on this knowledge.

## 2. The Evolution of Pathogen Perception by NLRs

**Figure 2:**
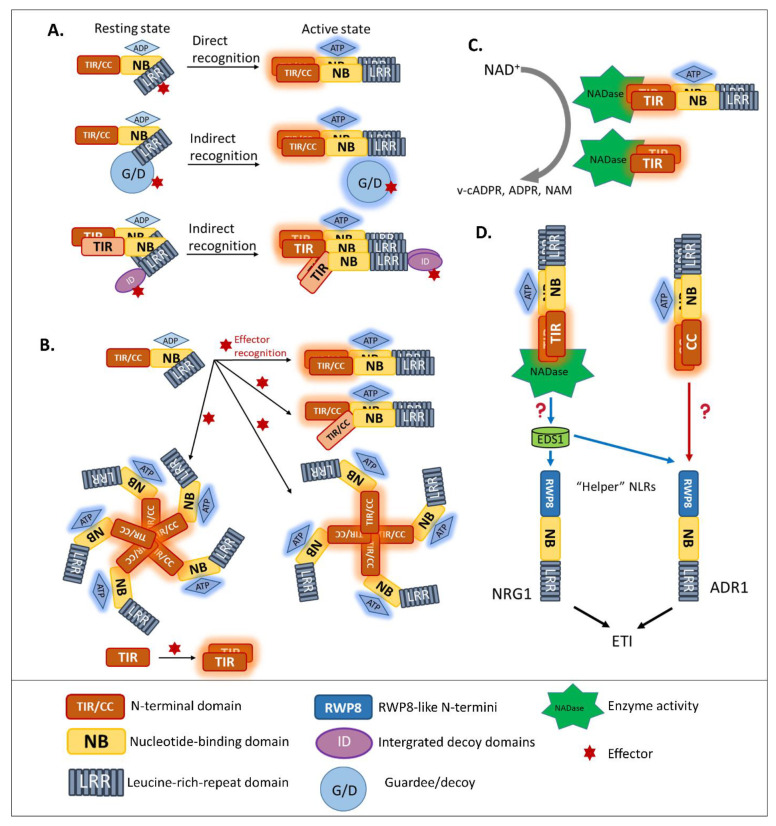
Diverse roles of nucleotide-binding and leucine-rich-repeat-containing receptors (NLRs) in immune signaling. (**A**) The evolution of NLR effector recognition systems. A common NLR consists of a diverse N-terminal domain, a central nucleotide-binding (NB) domain, and a C-terminal leucine-rich repeat (LRR) domain. NLRs are classified into two groups, depending on the N-terminus—toll interleukin-1-receptor (TIR) NLR (TNL) and coiled-coil (CC) NLR (CNL). NLRs recognize pathogen effectors directly through the LRR domain or indirectly through a host guardee/decoy protein. During coevolution, some NLRs acquired unusual integrated decoy (ID) domains for pathogen recognition. (**B**) The molecular switch of NLRs during effector recognition leads to NLR homo/hetero/oligomerization (the NLR “resistosome”). In response to pathogen effectors, the open-lid form of NLRs is formed. ADP–ATP exchange occurs, leading to NLR activation. The associations of NLRs, such as homodimerization, heterodimerization, and oligomerization, are important for downstream signaling. (**C**) After the formation of an NLR resistosome, the enzymatic activity of plant TIR produces nicotinamide adenine dinucleotide (NAD) derivatives. (**D**) Downstream components, ENHANCED DISEASE SUSCEPTIBILITY 1 (EDS1) and “helper NLRs” (N REQUIREMENT GENE 1 (NRG1) and ACTIVATED DISEASE RESISTANCE PROTEIN 1 (ADR1)), are required for NLR signaling. While CNLs depend on helper ADR1 to function (indicated by red arrow), TNLs activate NADase and require EDS1 (indicated by blue arrows) and both helpers (ADR1 and NRG1) for signal transduction. NRG1 and ADR1 mediate effector-triggered immunity (ETI) (indicated by black arrows). Question marks indicate the unknown mechanisms in NLR-triggered immunity.

As explained by the gene-for-gene hypothesis in which a resistance gene in the host plants corresponds to an avirulence (*avr*) gene in pathogens [40]; an NLR can recognize the presence of a pathogenic effector and trigger plant immunity. Effector recognition is described by diverse models explaining suggested direct or indirect detection [5,41]. In the earliest studies, cloning of *R* genes revealed the physical interaction of effectors with NLRs and the receptor–ligand model was proposed [5]. Direct effector recognition of NLRs depends on their LRR domains (Figure 2A).

Pathogen effectors evolutionarily adapted to avoid direct binding to NLRs, [42] while plant coevolution to restore direct effector detection seems to be slower. However, plants evolved indirect effector recognition systems (Figure 2A). To date, cases in which plants indirectly recognize effectors are more diverse and numerous than those illustrating direct recognition. In one indirect effector detection system—the guard model—NLRs perceive modifications of a host target protein, called the guardee [5]. One of the most famous examples of the guard model is the Arabidopsis RESISTANCE TO PSEUDOMONAS SYRINGAE PV. MACULICOLA 1 (RPM1)-INTERACTING PROTEIN4 (RIN4). RIN4—a negative regulator of immune responses—plays an important role in normal plant growth as a mutation of *RIN4* constitutively activates RESISTANT TO PSEUDOMONAS SYRINGAE 2 (RPS2) and is lethal [43,44]. When RIN4 is targeted by the *Pseudomonas syringae* effector proteins AvrRpm1 or AvrRpt2, it leads to the activation of the NLR RPM1 or RPS2, respectively [5,45,46]. In addition, the cleavage of RIN4 by AvrRpt2 leads to the activation of the NLR Malus x Robusta 5 in apples [47].

It is not easy for plant hosts to modify functional guardee proteins to further increase pathogen detection since pathogen effectors evolutionarily reduce the targeting of guardee proteins [42]. Plants, however, evolved an adapted recognition system that detects modified decoys. In the decoy model, plant R proteins recognize effector-mediated modifications of a plant decoy protein that has a very similar structure to the actual host target protein, thereby confining the pathogen effectors to the host recognition system [42]. For instance, the plant decoy protein, AVIRULENCE PROTEIN PSEUDOMONAS PHASEOLICOLA B (AVRPPHB)-SUSCEPTIBLE 1 (PBS1), is structurally similar to BOTRYTIS-INDUCED KINASE 1 (BIK1), a component of PTI that can be targeted and cleaved by effector AvrPphB to compromise resistance [48,49]. Cleavage of PBS1 by AvrPphB instead, triggers RPS5-mediated cell death [50,51,52,53].

By utilizing the indirect recognition systems, plants can likely expand the pathogen-recognition spectrum with a limited number of NLR proteins. For example, a pair of NLR, RESISTANCE TO RALSTONIA SOLANACEARUM 1 (RRS1)/RPS4, can recognize the presence of the *Ralstonia Solanacearum* effector *Pseudomonas*-out-protein P2 (PopP2), *Pseudomonas syringae* effector AvrRps4 and an unknown *Colletotrichum* effector [54,55]. In these recognition events, the decoy domain, containing the conserved amino acid sequence WRKYGQK found in WRKY transcription factors, became integrated into the NLR RRS1 during plant evolution. The WRKY decoy domain plays a critical role as a target of those effectors and triggers RRS1/RPS4-mediated immunity [56,57]. This integrated decoy model provides self-monitoring activity for NLRs in effector recognition (Figure 2A) [42]. It appears to be an effective tool for plants in detecting pathogen effectors, as shown by an examination of the canonical RRS1 and R-GENE ANALOG 5 (RGA5) homologs in other plant species. A wide range of integrated decoy types was found along with an abundance of NLRs with one or multiple integrated decoys [42,58]. Thus, the integrated decoy model highlights the structural evolution of plant NLRs in the context of pathogen evolution.

## 3. NLR Activation and Signaling Events Following Pathogen Recognition

### 3.1. Multi-Domain NLRs Act as Molecular Switches

A common NLR consists of a diverse N-terminal domain, an NB domain, and an LRR domain [42,48,59]. NLRs are normally classified into two groups (Figure 2A), depending on the N-terminus [48]. They are Toll interleukin-1-receptor NLRs (TNLs) and coiled-coil NLRs (CNLs). Other functional NLR-like proteins contain only a TIR or TIR-NB [42]. Each domain of the NLR has a function. The LRR domain is responsible for effector recognition and acts as an auto-inhibitory domain that prevents the auto-enabling downstream signaling [42,60]. The NB domain is specific to the ATP/ADP exchange and serves as a switch to turn NLRs on or off [42,59]. The phosphate-binding loop and methionine–histidine–aspartate region within the NB domain are two highly conserved motifs that essentially regulate the activity of NLRs [61]. While mutation of the phosphate-binding loop leads to NLR loss-of-function, the methionine–histidine–aspartate-motif mutation causes a gain-of-function [60,61]. The CC and TIR domains were originally thought to function as protein–protein interactions involved in NLR signaling [42].

In some cases, a common full-length NLR is not the only produced protein and alternative splicing can contribute to the generation of a truncated NLR [62,63,64]. For example, alternative splicing of the *RGA5* transcripts was identified [62]. In detail, unlike the alternative splicing isoform that lacks the integrated decoy (called the heavy metal-associated domain), fully-spliced RGA5 containing the integrated decoy domain confers resistance. Since the alternative RGA5 isoform does not contribute to resistance, the reason for *RGA5* alternative splicing remains unknown. However, *RPS4*, another example, was reported to show alternative splicing activity, which is crucial for the defense response [63]. Indeed, the removal of intron 2 or 3 or both in Landsberg *erecta RPS4* leads to a lack of resistance. In addition, TIR-NB RPS4 encoded from truncated Landsberg *erecta RPS4*, representing alternative splicing of *RPS4*, is insufficient to produce resistance. The experimental combination of both intron-deficient and truncated transcripts partially rescued AvrRps4-triggered resistance. This finding suggested that full-length genomic *RPS4*—which shows alternative splicing activity—is required [63]. Additionally, in this finding, the *RPS4* transcript isoforms in the combination were artificially functionally characterized, as compared to natural isoforms alternatively spliced from full-length genomic *RPS4*. This suggests that the ratio of alternative *RPS4* isoforms is critical for the AvrRps4 recognition. Taken together, in some cases, alternative splicing contributes to ETI by producing truncated NLRs. Nevertheless, how alternative NLRs function and the mechanism of the *R* gene alternative splicing remains an unexplained issue.

### 3.2. Homo/Hetero-Complex Formation Is Necessary for NLR Signaling

Previous studies reported that disruption of the Mildew A 10 (MLA10) CC dimerization abolished the activation of immunity [42,65,66], suggesting that CNLs require dimerization of the CC domain for signal transduction. Moreover, pentameric oligomerization of the CNL Hrp-dependent outer protein (Hop) Z-Activated Resistance 1, termed the “HopZ-Activated Resistance 1 resistosome”, is important for the formation of putative membrane pores and the immune response [67]. Similarly, several well-studied plant NLRs containing TIR domains, such as RECOGNITION OF PERONOSPORA PARASITICA 1 (RPP1), the flax resistance protein L6, RRS1, and RPS4, were found to require oligomerization by two distinct interfaces, for both self-association and defense signaling [68,69,70,71,72]. Similar to the case of MLA10, disrupting the homo-dimerization of L6 TIRs interferes with downstream signaling (Figure 2B). To effectively recognize the effector *Xanthomonas* outer protein Q (XopQ), the TNL Recognition of XopQ 1 resistosome requires tetramerization [73]. In addition, two asymmetric TIR homodimers that form an RPP1 tetrameric resistosome activate downstream signaling, in response to effector *Arabidopsis thaliana* Recognized 1 (Figure 2B) [69].

Hetero-associations in addition to homo-dimerization were proven to be an indispensable aspect in NLR-mediated signaling. Indeed, genetically-linked, paired NLRs were characterized as functioning together in conferring pathogen resistance [42]. RGA4/RGA5 is one of the functionally paired CNLs for *Magnaporthe oryzae* AVR-Pia/AVR-Pik-mediated resistance [41]. In addition, genetically-linked, paired TNLs, such as RPP2A/RPP2B, were found to provide resistance against *Hpa race Cala 2* [74], along with previously discussed RRS1/RPS4 recognize AvrRps4 and PopP2 [55,75]. In the paired cases listed above, one NLR, the “sensor NLR”, usually contains an evolutionarily incorporated integrated domain, and acts as an effector receptor, while the second NLR, the “executor NLR”, induces downstream signaling [41].

It is challenging to understand the function of truncated NLRs in plants. Expression of truncated RPS4, lacking NB-LRR domains but retaining the TIR with additional adjacent amino acids, can trigger immunity [75,76]. This indicates that the TIR of RRS1 is required for RPS4 auto-inhibition under normal conditions. Additionally, the TIR-only protein, Response to the bacterial type III effector protein HopBA 1 (RBA1), self-associates in response to effector HopBA1 (Figure 2B) [77], supporting the model that the RBA1 oligomerization induced by HopBA1 activates ETI. However, this self-association raises the question of how do NLR-like proteins lacking LRR or NB-LRR remain inactive in the absence of effectors? It could be that other components keep TNL-like proteins in inactive form. As in the auto-inhibition of the RPS4 TIR by the RRS1 TIR [76], the negative regulator for autoimmunity by a TIR-only protein might be another TIR-only protein or TNL. Further research is required to elucidate the plant immune response triggered by truncated NLRs.

### 3.3. Intramolecular Regulation of Guardee/Decoy Contributes to NLR-Mediated Resistance

It is now clear that R proteins can guard plant functions by monitoring different post-translational modifications of effector targets (guardee/decoy), and that different modifications can compete with or support each other. RIN4 was proposed to act as a phosphoswitch to detect the effector AvrRpm1. Targeting of RIN4 by AvrRpm1 causes the phosphorylation of threonine 166 within its C-terminal nitrate-induced domain; which leads to RPM1 activation and resistance [78]. A recent study revealed that the ADP-ribosylation of RIN4 at aspartate 153 by AvrRpm1, leads to threonine 166 phosphorylation and promotes RPM1 activation [79]. The addition of ADP-ribose supports the complete phosphorylation of threonine 166 in RIN4 [79]. Taken together, these reports indicate that several additive modifications can occur in a single guardee protein.

On the other hand, a post-translational modification of one effector target can antagonize another. The newest report of RRS1/RPS4-mediated immunity revealed that phosphorylation regulates the activation of paired RRS1/RPS4 [71]. In the absence of effector AvrRps4 or PopP2, phosphorylation at threonine 1214 in the integrated decoy WRKY domain keeps RRS1 from the resistant ecotype Wassilewskija, in a resting state. Dephosphorylation at that residue leads to the autoactivation of RRS1. Interestingly, PopP2 induces O-acetylation in the WRKY domain of RRS1, which competes with its phosphorylation and results in the dephosphorylated activated RRS1-mediated resistance to *Ralstonia Solanacearum* [71]. Other phosphorylation sites at the C terminus of RRS1 are required for PopP2 recognition, which enhances the interaction of the TIR domain with the WRKY domain. This study also proved that wild-type Columbia RRS1 lacks the C-terminal 83 amino acids that include the target phosphorylation sites, fails to recognize PopP2, and is thus susceptible to *Ralstonia Solanacearum*.

However, RRS1-mediated resistance to the *Pseudomonas syringae* effector AvrRps4 is determined by the association of the RRS1 C-terminus with its TIR, not by its phosphorylation status [71]. The C terminus and TIR of RRS1 interact with each other only in the presence of AvrRps4 [71]. During recognition of AvrRps4 or PopP2, the interaction of the RRS1 TIR domain with its C terminus is enhanced. This enhanced interaction releases the RPS4 TIR from the inhibition by the RRS1 TIR. Thus, the RPS4 TIR is activated, resulting in resistance to *Pseudomonas syringae*. The regulation of guardee/decoy monitoring is likely much more complex than is presently known. An additional 83 amino acids at the C-terminus of RRS1 from resistant ecotypes can function as a kinase docking site [71], suggesting that recruiting kinase protein activity or other post-translational modification could be involved in the RRS1/RPS4 activation.

### 3.4. News-Breaking: Enzyme Activity of Plant TIR in ETI Signaling

In animal immunity, an important function of Toll-like receptors is specifically recognizing their cognate pathogen-associated molecular patterns or synthetic compounds. Most animal Toll-like receptors contain two domains, one of which—the LRR domain—is necessary for PAMP recognition, while the other—the TIR domain—functions in signaling scaffolds. Some studies of animal-TIR domain crystallization showed that animal TIR associates during PAMP recognition. Animal TIR oligomerization is required for immune signaling, leading to the inflammatory cytokine response [80,81,82]. Unlike most Toll-like receptors, Sterile Alpha and TIR Motif Containing 1 (SARM1) was shown to have a surprisingly novel function [83,84]. Specifically, the nicotinamide adenine dinucleotide (NAD) hydrolase activity of its TIR domain contributes to axon degradation. This unique function raised the hypothesis that SARM1 probably arose from other domains in the animal system, through an evolutionary transfer event [85].

In plants, after NLR activation, the subsequent signal transduction cascade leading to the hypersensitive response and expression of plant immunity is at present unresolved. Although the signaling pathway of CNLs remains unclear, a piece of TNL downstream signaling was discovered [82,86]. As TIR domains are found in both plant intracellular TNLs and the animal cell surface Toll-like receptors, researchers compared the characteristics of plant TIR and animal TIR. Wan et al. and Horsefield et al. demonstrated that the TIR domains of plant TNLs are structurally similar to the TIR domain of mammalian SARM1 and that their enzymatic activity could degrade oxidized nicotinamide adenine dinucleotide (NAD^+^) (Figure 2C) [82,86]. Cell death activation and NAD^+^ catalytic activity of plant TIRs are self-association interface-dependent, placing the TIR enzyme activity downstream of TIR oligomerization. A conserved glutamic acid was found in plant TIR NAD^+^-cleaving enzymes and the human SARM1 NADase [86]. Although the putative catalytic glutamic acid does not affect the TIR association, it is the key residue for TIR-NADase activation. The accumulation of enzymatic products, such as variant-cyclic ADP-Ribose (v-cADPR), ADP-Ribose, and nicotinamide, which are necessary for immune signaling, are proposed to be downstream of TIR-enzyme activation. The NADase activity of the plant TIR domain is solely required for plant immunity, since the fusion of plant TIR (not animal or bacterial TIR) to the mammalian NLR Family CARD Domain Containing 4 activates immune signaling in plants [87]. Interestingly, in both *enhanced disease susceptibility 1* (*eds1*) and *n requirement gene 1* (*nrg1*) mutants, the activation of RBA1 accumulates v-cADPR but fails to induce a cell-death response [86], indicating that the accumulation of enzymatic products happens upstream of EDS1-NRG1. However, from catalytic product accumulation to EDS1-NRG1 downstream signaling, an undefined gap remains.

Even though plant TIR shows NADase activity, Wan et al. failed to observe significant NAD^+^ depletion in plants [86]. Apparently, the cleavage of NAD^+^ is tightly controlled and only occurs slightly during the immune response. The molecules generated from substrate digestion might activate the downstream components EDS1-NRG1 through some unknown receptor proteins, which recognize NAD^+^ cleavage products. Surprisingly, plant TIRs catalyze NAD^+^ to form v-cADPR, not the cADPR that is produced by SARM1. The v-cADPR also accumulates in Arabidopsis during HopBA1-mediated resistance [86]. These data suggest that among known NAD^+^ derivatives, v-cADPR currently could be considered a remarkable bio-marker for plant TIR-mediated immunity [85,86].

We expect that the discovery of homologous mammalian/plant TIR and enzymatic function of both would expand research. Currently, the chemical structure of v-cADPR remains unknown. Furthermore, it is unclear whether v-cADPR is the only biomarker and other unknown chemical products derived from NAD^+^ might also be involved in immune signaling. In addition, Ca^2+^ signaling, which is necessary for the hypersensitive response and other plant immune responses [88], could be controlled by NAD^+^ catalyzed products [89]. Thus, the linkage of Ca^2+^ and EDS1-NRG1 signaling, as well as the gap between TIR-NAD hydrolase activity and downstream EDS1-NRG1 are promising areas of research.

## 4. Helper NLR Cooperation beyond Genetically Linked Pairs

The concept of NLR cooperation broadened since more distinct NLRs were reported to be required for ETI, forming an NLR signaling network [42]. Some “sensor” NLRs, which recognize effectors, genetically interact with a limited number of “helper” NLRs that are required for cell death. These “helper” NLRs are required, not only for NLR-mediated effector recognition but also for signaling and programmed cell death (Figure 2D) [90,91]. In tomato and tobacco, CNL-type-NLRs required for cell death are crucial for NLR-triggered immunity [91].

Helper ACTIVATED DISEASE RESISTANCE PROTEIN 1 (ADR1)s and NRG1s were separated from CNLs into a new clade called Resistance to Powdery Mildew 8 (RPW8)-NLR (Figure 2D) [42]. The N-terminus of RPW8-NLR, with an atypical conserved R protein RPW8 that confers powdery mildew resistance [92], is crucial for the activation of downstream signaling. ADR1, ADR1-L1, and ADR1-L2 are three homologous RPW8-NLRs required for signaling of several NLRs, in resistance to bacterial or oomycete effectors [93,94]. The *adr1* triple mutant was shown to suppress the dwarf phenotype of autoimmune mutant *chilling sensitive 2*, *suppressor of npr1-1, constitutive1* (*snc1*), and *sensitive to low humidity 1*, which attenuate salicylic acid levels, and impair AvrRpt2- and AvrRps4-mediated immunity [93,94]. Although NRG1s are close homologs of ADR1s, they function independently [95]. NRG1 was first found to play a role downstream of the tobacco NLR protein N, which confers resistance to tobacco mosaic virus [96]. NRG1 also associates with EDS1 to recognize *Xanthomonas* effector XopQ in XopQ-mediated resistance in tobacco [97]. Interestingly, NRG1s cannot be found in plants lacking TNL, suggesting that they might function in TNL-mediated immunity [95]. Arabidopsis full-length NRG1A and NRG1B, but not truncated NRG1C, have a redundant function in *chilling sensitive 3*-triggered autoimmunity [95]. In detail, the *nrg1s* null mutant can convert the dwarfism of autoimmune *chilling sensitive 3* to a normal phenotype.

Downstream of NLR-triggered immunity, ADR1s and NRG1s, function synergistically in Arabidopsis [95]. For instance, CNL RPS2, TNL RPP2, RPP4, and paired NLR RRS1/RPS4 were reported to signal via helper ADR1s [93]. A further study demonstrated that entire RPP2-, RPP4-, and RRS1/RPS4-mediated immune responses require helper NRG1s [98]. Therefore, these TNLs transduce the signal via ADR1s, as well as NRG1s. However, no study showed physical interactions of ADR1s with other “sensor” NLRs or downstream proteins. Arabidopsis NRG1s also do not interact with themselves or other functional known proteins, downstream of TNLs [95]. Therefore, the process through which NRG1s and ADR1s trigger NLR-mediated immune response is still an unresolved question.

## 5. Transcriptional Control in ETI

### 5.1. NLRs Regulate Transcriptional Control

In some situations, the interaction of an NLR with a transcriptional regulator was observed [99,100]. For instance, the TNL SNC1 interacts with TOPLESS-RELATED PROTEIN 1 (TPR1), a positive regulator that suppresses the known negative regulators [101]. SNC1 also complexes with basic Helix-Loop-Helix 84 (bHLH84), which positively regulates immunity [102]. Interestingly, bHLH84 associates not only with SNC1 but also with the TNL RPS4. Overexpression of *bHLH84* accelerates resistance, while the double mutant of *bHLH84* and its paralog-*bHLH54* compromises RPS4 and SNC1 immunity [102]. Another example is barley CNL MLA10, which can interact with two antagonizing transcriptional factors, WRKY1 and Myeloblastosis (MYB) 6 [100]. Association of MLA10 with MYB6 promotes MYB6 DNA binding and positively regulates the immune response. Additionally, MLA10 disrupts the WRKY1–MYB6 complex, thus freeing MYB6 from WRKY1 repression and permitting it to bind DNA.

An interesting transcriptional regulation of ETI is exemplified by the SUPPRESSOR OF rps4-RLD1 (SRFR1)–NLR interaction. SRFR1 functions as a negative regulator of AvrRps4- and HopA1-triggered immunity, and acts as an adaptor protein that interacts with TNLs, such as RPS4 and RPS6, and with EDS1 [103,104,105,106]. SRFR1 contains a Tetratricopeptide Repeat domain that shows sequence similarity to that of *Saccharomyces cerevisiae* Suppressor of *sucrose-nonfermenting1* 6, which functions as a transcriptional repressor. Increased transcript levels of defense-related genes are induced in *srfr1* mutants [107]. In addition, SRFR1 physically interacts with members of the Teosinte branched1/Cincinnata/Proliferating transcription factor family [108]. This suggests that SRFR1 also functions as an adaptor protein that negatively regulates ETI-associated transcriptional plant immune responses. Along with regulating SRFR1 interactions with TNL and immune transcriptional control, TPR2, unlike TPR1, was shown to inhibit SNC1 auto-activation [109]. The interaction of TPR2 with TPR1 and SNC1 was thought to weaken the SNC1–TPR1 association, resulting in an indirect negative effect on *SNC1* expression [109].

### 5.2. Effector-Regulated Transcriptional Control

Transcription of activator-like effectors from *Xanthomonas* spp. can mimic eukaryotic transcription factors by directly regulating gene expression in plants [48,100]. For example, effector AvrXa7 targets the effector binding elements in the promoter of the rice gene *SWEET14*, to induce sugar flux for pathogen fitness [48]. Through evolution, plants integrated effector binding elements into the promoters of executor *R* genes, providing a powerful strategy for pathogen resistance. *Xanthomonas* transcription activator-like effector AvrXa27 targets the effector binding elements of the rice *R* gene *Xa27*, inducing *Xa27* expression and subsequent elicitation of the hypersensitive response. In addition, the *Xanthomonas* transcription activator-like effector AvrBs3 directly increases *up-regulated by AvrBs3 20* gene expression in susceptible pepper, for pathogen advantage. However, in resistant pepper, the *R* gene *bacterial spot3* containing the up-regulated by AvrBs3 box is an executor gene that intercepts AvrBs3, thus converting virulence to avirulence [100].

## 6. PTI/ETI Unity Produces Full Plant Immunity

**Figure 3:**
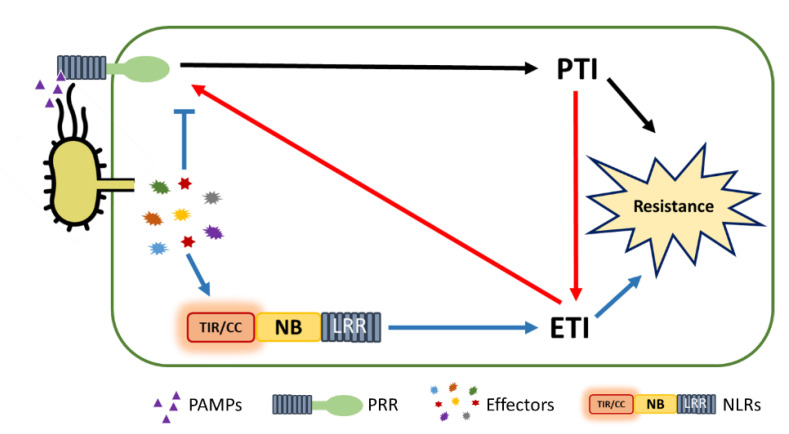
The integration of pattern-triggered immunity (PTI) and effector-triggered immunity (ETI) in plant immunity. In response to pathogens, an induced defense response is turned on by pattern recognition receptors (PRRs)-mediated PTI (indicated by black arrows) and nucleotide-binding and leucine-rich-repeat-containing receptors (NLRs)-mediated ETI (indicated by blue arrows). Recent reports indicate that there is substantial crosstalk between PTI and ETI (indicated by red arrows). ETI functions through PTI components and potentiates PTI signaling. Synergistically, PTI also enhances the ETI response. The cooperation of PTI and ETI mutually contributes to plant innate immunity.

It was always clear that PTI functions as the first tier in an induced defense against pathogens. However, ETI apparently functioned only after an effector had suppressed PTI. This led to the interpretation that PTI had little effect on the immune response during ETI. However, increasing evidence indicates that PTI and ETI signaling interact (Figure 3). Transcriptional profiling of PTI and ETI largely overlap [36,110]. In addition, Hatsugai et al. showed that PTI suppresses an ETI signaling sector, suggesting that PTI controls the immune signal pathways to fine-tune plant defenses and limit useless fitness costs [111].

Recently, two independent research groups presented evidence suggesting that NLR-mediated plant immune responses require PRRs to function [38,39]. Specifically, two PTI-related mutants, *bbc* and *fec*, were compromised in effector AvrRpt2-mediated resistance, while in Col-0, AvrRpt2-triggered immunity was increased, in response to PAMP flagellin peptide flg22 [39]. This suggested that PTI signaling through PRR/co-receptors induces ROS accumulation during an ETI response. The research group also found that the enhancement of ROS in ETI was due to β-nicotinamide adenine dinucleotide phosphate oxidase activity. Indeed, *RESPIRATORY BURST OXIDASE HOMOLOGUE D* (*RBOHD*), a canonical pathogen-triggered ROS gene, regulates ROS produced by ETI. Furthermore, the well-known PTI-defective mutant, *rbohd*, exhibited a susceptible phenotype in response to *Pseudomonas syringae* DC3000 expressing *avrRpt2*. This data suggested that RBOHD acts as a central hub that links PTI and ETI. In detail, RBOHD only produces ROS when it is phosphorylated by BIK1, emphasizing the importance of the PTI protein kinase BIK1 in an ETI response. Additionally, Ngou et al. found considerable accumulation of PTI-responsive gene transcripts, as well as PTI-related proteins, by conditionally expressing effector AvrRps4 in Arabidopsis [38]. With bacterial treatment, Yuan et al. obtained similar results in which PTI components such as BRASSINOSTEROID INSENSITIVE 1-associated receptor kinase 1, BIK1, mitogen-activated protein kinase 3, and mitogen-activated protein kinase 6, were boosted by AvrRpt2-triggered immunity [39]. Taken together, these results indicate that ETI signals through PTI and increases the PTI response (Figure 3). Concurrently, PTI also enhances ETI and is functionally essential for the ETI response (Figure 3). The synergistic cooperation of PTI and ETI provides a robust immunity to confront pathogenic invasion. In particular, PTI combats pathogenic microbes by reinforcing cell walls, increasing callose deposition, and producing anti-microbial compounds. Meanwhile, ETI sharpens PTI function by upregulating the PTI components. These findings revealed the mutual relationships of immune extra- and intracellular receptors, providing insight into the whole picture of plant immunity. However, the mechanism of how ETI potentiates PTI remains a question to explore. The biochemical function of NLR downstream components, such as helper NLRs, EDS1, and NON-RACE-SPECIFIC DISEASE RESISTANCE 1, is also still unclear, preventing the determination of component positions in PTI–ETI crosstalk.

## 7. Perspectives

Plant pathogens consistently evolve to bypass host immunity [112]. Knowing the enemy (pathogens) and embracing friends (plants) are important for agriculture and our survival. To date, many ETI components and regulation modes were studied, providing the different puzzle pieces of knowledge that reveal how plants overcome pathogen invasion and develop resistance. By applying the accumulated information and newly developed techniques, we expect a bright future of engineered crops with broad-spectrum resistance.

There are several methods to generate resistant crops. One method, traditional breeding, not only requires a very long time to generate a resistant cultivar but also entails the challenge of transferring the resistant allele from one plant species to another. Alternatively, gene transfer can be utilized for the introgression of immune receptors across plants. The Arabidopsis PRR, which recognizes PAMP Elongation-Factor thermal unstable, successfully conferred broad-spectrum bacterial resistance in tomatoes [113], but the crop, in this case, would be considered to be a genetically modified organism and would face regulatory issues.

In the case of NLRs, it is not easy to expand the range of effector recognition, since NLR-mediated resistance is quite specific. It is possible to generate gain-of-function plants by mutating NLRs or other immune components. The criteria for success are that mutation does not cause any growth/defense trade-off phenotype, and stabilizes crop production. Some studies successfully expanded NLR recognition through random mutation in the NB-LRR domains [42]. However, those random mutations occur case-by-case, providing no principle for applying the gain-of-function to the many types of NLRs in a variety of plants.

Alternatively, modifying integrated decoy NLRs to recognize novel effectors is a very promising method for a new era [42,61]. Indeed, PBS1 decoy engineering appears to be a fascinating strategy in which the engineered PBS1 expanded effector recognition by NLR RPS5 [51]. Identifying ortholog proteins in the vast data banks that now exist also provides the opportunity to apply recognition systems that originate from model plants. Indeed, the proof of principle of the PBS1 decoy system was shown in soybean [114], promising the possible application of resistance engineering to other crop plants. Additionally, the PBS1 decoy engineering provided an insight into expanding effector recognition that depends on the modification of a decoy through effector protease activity. Protease activity was observed not only in AvrPphB, but also in other bacterial effectors, such as AvrRpt2 [43] and HopB1 [115], and in many plant potyviruses. Based on the known cleavage activity of virulence factors, introducing the chimera of an NLR-cognate positive/negative regulator, with the effector cleavage site in between, appears to be a promising strategy.

In another approach, a recent study presented a new method to control the translational level of immune components to engineer resistant crops [116]. Since ETI potentiates PTI and some PTI components are required in ETI response [38,39], these components can probably be effective targets for translational control in engineering. However, to be effective, more knowledge is required to determine the key nodes of PTI–ETI crosstalk and how changes in the relative expression of components impact the outcomes of yield and resistance.

Along with the gene-transfer method, Clustered Regularly Interspaced Short Palindromic Repeats is now a powerful tool for genome editing for producing resistant phenotypes. Using this tool to edit the virulence target of a bacterial effector, fire blight susceptibility in apple was reduced [117]. In rice, five mutations in the promoters of three sucrose *SWEET* transporter genes conferred broad-spectrum resistance to bacterial blight [118]. This research suggests that common targets of effectors can be potential candidates for gain-of-resistance genome editing. Additionally, today, it is possible to excise the exogenous DNA from an engineered crop [117], so that it is not considered to be a genetically modified organism.

As seen in the above examples, our present understanding of NLRs and other immune components is being applied to increase plant resistance to pathogens. Although hidden pieces in the NLR-mediated immunity signaling pathway require further study, we predict that more promising strategies are certain to come on the scene for future crop protection.

## Abbreviations

ADR1	ACTIVATED DISEASE RESISTANCE PROTEIN 1
Avr	Avirulence
AvrPphB	Avirulence protein Pseudomonas phaseolicola B
bHLH84	basic Helix-Loop-Helix 84
BIK1	BOTRYTIS-INDUCED KINASE 1
CC	Coiled-Coil
CNL	Coiled-coil NLR
DAMP	Damage-Associated Molecular Pattern
EDS1	ENHANCED DISEASE SUSCEPTIBILITY 1
ETI	Effector-Triggered Immunity
Hop	Hrp-dependent outer protein
HR	Hypersensitive Response
ID	Integrated Decoy
LRR	Leucine-Rich Repeat
MAPK	Mitogen-Activated Protein Kinase
MLA10	Mildew A 10
MYB	Myeloblastosis
NAD	Nicotinamide Adenine Dinucleotide
NB	Nucleotide-Binding
NLR	Nucleotide-binding and Leucine-rich-repeat-containing Receptor
NRG1	N REQUIREMENT GENE 1
PAMP	Pathogen-Associated Molecular Pattern
PBS1	AVRPPHB-SUSCEPTIBLE 1
PopP2	*Pseudomonas*-out-protein P2
PRR	Pattern Recognition Receptor
PTI	Pattern-Triggered Immunity
R	Resistance
RBA1	Response to the bacterial type III effector protein HopBA1
RBOHD	RESPIRATORY BURST OXIDASE HOMOLOGUE D
RGA5	R-GENE ANALOG 5
RIN4	RPM1-INTERACTING PROTEIN 4
ROS	Reactive Oxygen Species
RPM1	RESISTANCE TO PSEUDOMONAS SYRINGAE PV. MACULICOLA 1
RPP1	RECOGNITION OF PERONOSPORA PARASITICA 1
RPS2	RESISTANCE TO PSEUDOMONAS SYRINGAE 2
RPW8	Resistance to Powdery Mildew 8
RRS1	RESISTANCE TO RALSTONIA SOLANACEARUM 1
SARM1	Sterile Alpha and TIR Motif Containing 1
SNC1	SUPPRESSOR OF npr1-1, CONSTITUTIVE1
SRFR1	SUPPRESSOR OF rps4-RLD1
TIR	Toll-interleukin 1-like Receptor
TNL	Toll interleukin-1-receptor NLR
TPR1	TOPLESS-RELATED PROTEIN 1
v-cADPR	Variant-cyclic ADP-ribose
XopQ	*Xanthomonas* outer protein Q
